# EEG Microstates Indicate Heightened Somatic Awareness in Insomnia: Toward Objective Assessment of Subjective Mental Content

**DOI:** 10.3389/fpsyt.2018.00395

**Published:** 2018-09-06

**Authors:** Yishul Wei, Jennifer R. Ramautar, Michele A. Colombo, Bart H. W. te Lindert, Eus J. W. Van Someren

**Affiliations:** ^1^Department of Sleep and Cognition, Netherlands Institute for Neuroscience (NIN), An Institute of the Royal Netherlands Academy of Arts and Sciences, Amsterdam, Netherlands; ^2^Bernstein Center Freiburg and Faculty of Biology, University of Freiburg, Freiburg, Germany; ^3^Centre for Chronobiology, Psychiatric Hospital of the University of Basel (UPK), Basel, Switzerland; ^4^Department of Psychiatry, Amsterdam UMC, Vrije Universiteit Amsterdam, Amsterdam, Netherlands; ^5^Department of Integrative Neurophysiology, Center for Neurogenomics and Cognitive Research (CNCR), Amsterdam Neuroscience, Vrije Universiteit Amsterdam, Amsterdam, Netherlands

**Keywords:** high-density EEG, resting state, microstate, insomnia disorder, wakefulness, mental content, somatic awareness, electrical neuroimaging

## Abstract

People with Insomnia Disorder (ID) not only experience abundant nocturnal mentation, but also report altered spontaneous mental content during daytime wakefulness, such as an increase in bodily experiences (heightened somatic awareness). Previous studies have shown that resting-state EEG can be temporally partitioned into quasi-stable microstates, and that these microstates form a small number of canonical classes that are consistent across people. Furthermore, the microstate classes have been associated with individual differences in resting mental content including somatic awareness. To address the hypothesis that altered resting mental content in ID would be reflected in an altered representation of the corresponding EEG microstates, we analyzed resting-state high-density EEG of 32 people with ID and 32 age- and sex-matched controls assessed during 5-min eyes-closed wakefulness. Using data-driven topographical k-means clustering, we found that 5 microstate classes optimally explained the EEG scalp voltage map sequences across participants. For each microstate class, 3 dynamic features were obtained: mean duration, frequency of occurrence, and proportional coverage time. People with ID had a shorter mean duration of class C microstates, and more frequent occurrence of class D microstates. The finding is consistent with previously established associations of these microstate properties with somatic awareness, and increased somatic awareness in ID. EEG microstate assessment could provide objective markers of subjective experience dimensions in studies on consciousness during the transition between wake and sleep, when self-report is not possible because it would interfere with the very process under study. Addressing somatic awareness may benefit psychotherapeutic treatment of insomnia.

## Introduction

Insomnia Disorder (ID) is a chronic disorder characterized by both nighttime and daytime symptoms. Nighttime symptoms include difficulty falling asleep, frequent or prolonged awakenings during the night, and early morning awakening. Daytime symptoms refer to fatigue, impaired concentration, mood disturbances, or other subjective complaints on daytime functioning (1). The maintenance of insomnia symptoms is likely to involve a host of cognitive factors including various forms of spontaneous mental activity as well as dysfunctional beliefs and attentional biases (2–4). A recent study found that people suffering from ID markedly differ from those without sleep complaints in several dimensions of spontaneous awareness, thoughts, and feelings (5) quantified using the Amsterdam Resting State Questionnaire (ARSQ) (6). The neural bases of the altered cognitive processes in ID are currently not well understood. As subjective mental states are increasingly viewed as arising from the interactions between distributed brain networks (7–9), studying the collective dynamic organization of brain network activity might reveal key mechanisms underlying the altered awareness, thoughts, feelings, and other mental states in ID.

Electroencephalography (EEG) is a relatively cost-efficient and non-disruptive means to measure brain activity and has been widely utilized in research on mental processes. EEG microstate analysis is a particularly valuable methodology for quantifying the rapid dynamics of large-scale brain networks not captured by the limited temporal resolution of functional magnetic resonance imaging (fMRI) (10, 11). EEG microstates are defined as quasi-stable scalp voltage configurations which on average last for tens of milliseconds. Transitions between microstates are assumed to reflect dynamic activation of distributed brain networks at sub-second timescales (12, 13). Resting-state EEG microstates during eyes-closed wakefulness are most commonly grouped into 4 classes (conventionally labeled as microstate classes A, B, C, and D) through topographical clustering techniques (10), although in a recent study up to 7 distinct microstate classes were identified (14). Combined EEG-fMRI has been utilized to confirm that the blood-oxygen-level dependent (BOLD) correlates of the 4 canonical microstate classes exhibit spatial patterns of well-known resting-state networks (15). Specifically, intra-individual fluctuations of class A, B, C, and D microstates were linked to activation of the “auditory,” “visual,” “salience,” and “attention” networks, respectively. In addition, studies using electric source imaging have provided complementary information about the neural substrates of EEG microstates, such as sources common to all microstates which cannot be detected with BOLD fMRI (14, 16).

Although EEG microstates have been hypothesized to represent the building blocks of mentation, or “atoms of thoughts and emotions” (17), efforts to directly test the relationship between microstate properties and subjective mental content have only recently emerged. An experimental study adopting a within-subjects task manipulation reported increased presence of class A and B microstates while participants were engaged in visual and verbal thinking tasks, respectively (18). A second study correlated microstate properties during the eyes-closed resting state with each dimension of the ARSQ across participants (19). The most robust finding of this study was a negative association between the proportional coverage time of class C microstates and the “somatic awareness” dimension of the ARSQ. The mean duration of class C microstates also showed a negative association with somatic awareness. In addition, the proportional coverage time and mean duration of class B microstates were positively associated with the “comfort” dimension of the ARSQ. Properties of class D microstates showed rather nonspecific associations with multiple ARSQ dimensions, while those of class A microstates were not systematically associated with any specific ARSQ dimension.

Given these observed associations between subjective mental content and microstate properties during the eyes-closed wake resting state, we hypothesized that people with ID would exhibit altered microstate dynamics in line with their altered resting mental content (5). The present study utilized 256-channel high-density EEG (HD-EEG) in a sample of 32 patients and 32 matched controls to verify the hypothesis. To our knowledge, this is the first study on EEG microstate dynamics in ID.

## Materials and methods

### Participants and EEG recordings

We analyzed resting-state HD-EEG recordings of 32 people meeting the DSM-5 (20) criteria for ID (25 female, age range 21–67 y) and 32 age- and sex-matched controls (CTRL) without sleep complaints (26 female, age range 22–70 y) from a previously reported study (21). Participants were recruited through advertisement and the Netherlands Sleep Registry and were screened by telephone followed by a face-to-face structured interview. Exclusion criteria for all participants were: (1) diagnosed sleep apnea, restless legs syndrome, narcolepsy, or other somatic, neurological, or psychiatric disorders; (2) use of sleep medications within the prior 2 months; (3) overt shifted or irregular sleep–wake rhythms, assessed using 1 week of actigraphy (Actiwatch AW4, Cambridge Neurotechnology Ltd., Cambridge, United Kingdom or GENEActiv Sleep, Activinsights Ltd., Kimbolton, United Kingdom) supplemented by sleep diaries; (4) scores above the minimal to mild range of anxiety or depression symptom severity, as evaluated by either the Hospital Anxiety and Depression Scale (HADS) (22), or the Beck Anxiety Inventory (BAI) (23) and Beck Depression Inventory (BDI-IA) (24). Furthermore, all patients had Insomnia Severity Index (ISI) (25) scores above 10, and all controls had ISI scores less than 8. The study was approved by the ethics committee of the VU University Medical Center, Amsterdam, The Netherlands. All participants provided written informed consent.

HD-EEG was acquired in a laboratory setting using a 256-channel HydroCel Geodesic Sensor Net (Electrical Geodesic Inc., Eugene, OR) connected to a Net Amps 300 amplifier (input impedance: 200 MΩ, A/D converter: 24 bits), with the ground electrode placed at the centro-parietal midline and reference at the vertex. Electrode impedances were kept below 100 kΩ. Signals were online band-pass filtered between 0.1–100 Hz and digitized at 1000 Hz.

### Protocol

On the recording days, participants were asked to refrain from alcohol and drugs, as well as to limit consumption of caffeinated beverages to a maximum of 2 cups, which were allowed only before noon. Wake resting-state HD-EEG was recorded during the evening (between 19:00 and habitual bedtime) while the participant was seated upright. The original protocol consisted of eyes-open (EO) followed by eyes-closed (EC) conditions of 5-min duration each (21). Vigilance level was monitored in real-time during recording by laboratory staff. In the occasional cases where signs of falling asleep were observed (e.g., slow eye movements, attenuation of alpha waves), the participant was alerted and recording of the 5-min assessment was restarted. Since so far validation of the reliability of microstate properties has been carried out only in EC (11), and only the links between mental content and microstate properties during EC have been demonstrated (18, 19), we here restricted analyses to the EC data.

### EEG preprocessing

Preprocessing and signal analyses were performed in MATLAB 8.3 (The Mathworks Inc., Natick, MA). EEG data were preprocessed using the MEEGPIPE toolbox (https://github.com/meegpipe/meegpipe). The preprocessing procedure involved several automatic and manual steps as detailed previously (21). Briefly, voltage drifts within channels were estimated by local polynomial approximation (26) and subtracted. The signals were downsampled to 250 Hz and band-pass filtered at 0.5–62.5 Hz. Modified *z*-score (27) criteria applied to the standard deviation, range, and gradient of the voltage signals were used to marked noisy EEG channels and sporadic noisy segments. Noisy channels were linearly interpolated from neighboring channels. Sporadic noisy segments were excluded from analyses. The remaining segments were submitted to independent component decomposition (28). Components of power-line noise, eye movement, pulse wave, and cardiac field artifacts were identified through visual inspection of their time courses and topographical distribution and projected out of the data. The total duration of artifact-free data did not differ between groups (mean ± standard deviation: ID = 289.4 ± 50.2 s, CTRL = 289.2 ± 21.4 s, *p* = 0.98).

### Microstate analysis

The method for identifying the microstate classes closely followed previous studies that used the same 256-channel HydroCel Geodesic Sensor Net (29, 30). The preprocessed EEG signals from 204 electrodes overlying the scalp area (excluding electrodes at the cheeks and the nape) were further band-pass filtered at 1–40 Hz and re-referenced to the common average. Momentary topographies at the local maxima of the global field power (GFP) were submitted to k-means clustering based on their absolute spatial correlations (ignoring polarity differences). The k-means clustering routine was run multiple times for each participant with the pre-defined number of clusters varying from 3 to 11. The optimal number of clusters for each individual, $k_{i}^{*}$, was then determined by the Krzanowski–Lai criterion which identifies the point of maximal normalized curvature on the dispersion curve (31). The normalized cluster-mean topographies from all participants were then submitted to group-level k-means clustering, which was also run multiple times with the pre-defined number of clusters varying from 3 to 11. A constraint was imposed such that the $k_{i}^{*}$ clusters from an individual had to be assigned to $\min\left(\overset{*}{\underset{i}{k}},k\right)$ distinct group-level classes, where *k* is the pre-defined number of group-level clusters (viz. classes) in a particular run. Finally, the Krzanowski–Lai criterion was applied again to determine the optimal number of group-level clusters, k^*^.

The average topographies of the group-level microstate classes were fitted back to individual EEG recordings competitively. Each momentary topography at the GFP local maxima was assigned to the microstate class with which the highest absolute spatial correlation was attained. Consecutive GFP local maxima assigned to the same microstate class were merged into one microstate, with start and end times of each microstate defined as midpoints to the neighboring GFP local maxima (11, 18). Microstates whose start or end times could not be estimated (i.e., those at the very beginning and very end of the recording and those bordering noisy segments) were omitted from analyses. From the resulting sequences of alternating microstates, we calculated the following standard dynamic features for each microstate class for each participant (11, 12, 19): (1) Mean Duration—the mean duration in milliseconds of the microstates of a particular class. (2) Frequency of Occurrence—the number of microstates of a particular class per second. (3) Proportional Coverage Time—the percentage of time spent in a particular microstate class.

It has been suggested that fitting microstate classes only at the GFP local maxima, thus ignoring the fine-grained dynamics between GFP local maxima, might be suboptimal (13). However, we found that almost perfectly correlated feature values were produced by fitting microstate classes either only at the GFP local maxima or at every timeframe (Supplementary Table 1). In other words, fitting microstate classes at either timescale provides essentially equivalent information, at least as far as the dynamic features we studied here are concerned.

### Statistical analyses

Randomized permutation tests for topographical differences (conventionally referred to as TANOVA) (11, 31) were used to compare microstate topographies between ID and CTRL. Group differences in microstate dynamic features (mean duration, frequency of occurrence, and proportional coverage time for each class) were expressed in Cohen's *d* and their significance was further assessed by means of linear regression modeling with two-tailed Wald *z*-tests, performed using R (32). Linear mixed-effects regression models with Gaussian random effects (33) were set up for mean duration and frequency of occurrence and Dirichlet regression (34, 35) for proportional coverage time, as the latter better models compositional data which sum up to 100% within each participant.^1^ All regression models included age and sex as covariates in addition to group contrasts and the within-subjects factor (microstate class). In total, 3k^*^ group effects were tested (3 dynamic features for each of the k^*^ identified microstate classes). Following Rieger et al. (36), the group contrasts for proportional coverage time were not considered independent tests because the values of proportional coverage time could be deduced from mean duration and frequency of occurrence. Therefore, the *p*-value threshold $\frac{0.05}{\left(2k^{*}\right)}$ for controlling the family-wise error rate (FWER) was employed.

## Results

### Demographic and clinical characteristics

Demographic and clinical characteristics of patients and controls are summarized in Table 1. As expected, patients had significantly higher ISI scores and tended to report higher BAI, BDI-IA, and HADS scores than controls.

**Table 1:** Characteristics of participants (mean ± standard deviation).

	**Control (*n* = 32)**	**Insomnia disorder (*n* = 32)**	***p***
Age, y	46.8 ± 15.0	48.5 ± 14.1	0.64
Sex, female/male	26/6	25/7	1
ISI	2.00 ± 1.97	17.19 ± 3.75	<0.0001
BAI	2.00 ± 2.33	6.42 ± 4.93	0.06
BDI-IA	2.00 ± 1.85	4.75 ± 3.84	0.11
HADS—Anxiety	4.33 ± 2.10	5.75 ± 2.38	0.06
HADS—Depression	1.88 ± 1.65	3.60 ± 3.30	0.09

*ISI, Insomnia Severity Index; BAI, Beck Anxiety Inventory; BDI-IA, Beck Depression Inventory IA; HADS, Hospital Anxiety and Depression Scale*.

*p-values are determined by Fisher exact test for sex and by Wilcoxon rank-sum tests for the other variables*.

### Microstate topographies

**Figure 1:**
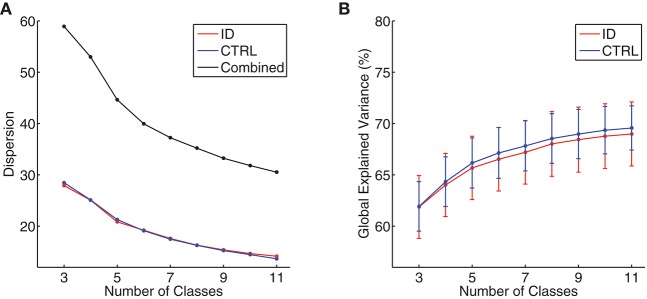
**(A)** Group-level dispersion (i.e., within-cluster global dissimilarity) for 3- to 11-class models, displayed separately for people with Insomnia Disorder (ID, red line), healthy controls (CTRL, blue line), and all participants combined (black line). **(B)** Mean percentages of global variance explained by all microstate classes when microstate topographies resulting from 3- to 11-class models were fitted back to individual EEG data, displayed separately for people with Insomnia Disorder (ID, red line) and healthy controls (CTRL, blue line). Error bars indicate 95% confidence intervals.

At the individual level, the Krzanowski–Lai criterion suggested 4–6 as the optimal number of clusters for all participants (mean ± standard deviation: ID = 4.38 ± 0.55, CTRL = 4.31 ± 0.47, *p* = 0.63). At the group level, the Krzanowski–Lai criterion suggested a 5-class model as optimal either for ID, for CTRL, or for all participants combined. Figure 1 shows the overall dispersion (i.e., within-cluster global dissimilarity) for different numbers of group-level clusters, as well as the corresponding mean percentages of global variance explained when the cluster topographies were fitted back to the GFP local maxima in individual EEG recordings. The global explained variance derived from the optimal 5-class model did not differ significantly between the two groups (mean ± standard deviation: ID = 65.68 ± 8.57 %, CTRL = 66.16 ± 6.77 %, *p* = 0.80).^2^

**Figure 2:**
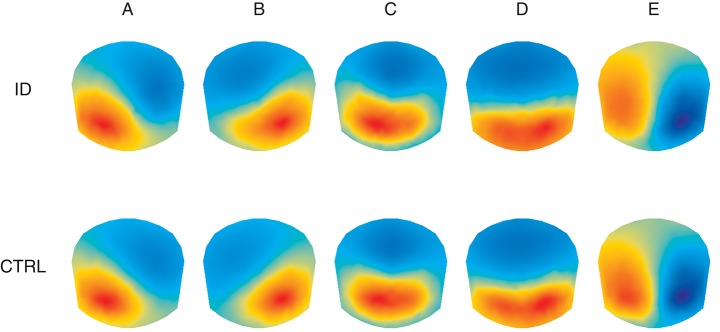
Average topographic maps for the 5 optimal microstate classes **(A–E)** in people with Insomnia Disorder (ID) and healthy controls (CTRL). Note that by convention microstate labeling only depends on the spatial configuration while the absolute voltage and polarity are ignored.

Figure 2 shows the average topographies for the 5 identified microstate classes in both groups. The average topographies for 4 of the identified classes resemble the 4 canonical microstate topographies reported in previous studies and are labeled hereafter as microstate classes A, B, C, and D accordingly. The 5th microstate class resembles microstate class E identified by Custo et al. (14) in a large sample and therefore we also label it as microstate class E. Permutation TANOVA performed separately for each microstate class revealed that the microstate topographies significantly differed between the two groups for microstate class A (*p* = 0.04) but not for the other classes (all *p* > 0.15).

### Microstate dynamics

The mean and standard deviation of mean duration, frequency of occurrence, and proportional coverage time for each microstate class for each group are presented in Table 2. Linear mixed-effects regression indicated group differences in the mean duration of class C microstates (group effect ± standard error = −7.61 ± 2.68 ms, *z* = −2.84, *p* = 0.0045) and the frequency of occurrence of class D microstates (group effect ± standard error = 0.62 ± 0.26 s^−1^, *z* = 2.38, *p* = 0.018) were significant at the *p* < 0.05 level, albeit only the former had a *p*-value below the FWER-controlling threshold (0.05/10 = 0.005). Both differences had medium unadjusted effect sizes (Cohen's *d* = −0.57 and 0.47, respectively). Other group effects on mean duration or frequency of occurrence did not reach significance (all *p* > 0.18). Dirichlet regression revealed no significant group effect on proportional coverage time for any of the microstate classes (all *p* > 0.10), although the differences for microstate classes C and D were of medium unadjusted effect sizes (Cohen's *d* = −0.42 and 0.51, respectively).

**Table 2:** Microstate dynamic features (mean ± standard deviation).

	**Microstate class**	**Control (*n* = 32)**	**Insomnia disorder (*n* = 32)**	**Cohen's *d***	***p***
Mean Duration (ms)	A	53.88 ± 7.84	51.45 ± 8.62	−0.30	0.45
	B	57.55 ± 11.02	53.54 ± 7.89	−0.42	0.18
	C	60.71 ± 16.74	52.69 ± 11.09	−0.57	0.0045
	D	60.40 ± 10.60	62.67 ± 15.43	0.17	0.31
	E	50.47 ± 7.58	47.30 ± 6.69	−0.44	0.30
Frequency of Occurrence (1/s)	A	3.42 ± 0.87	3.64 ± 0.80	0.26	0.47
	B	3.71 ± 0.45	3.89 ± 0.86	0.26	0.56
	C	3.67 ± 1.18	3.51 ± 1.41	−0.12	0.47
	D	3.80 ± 1.61	4.45 ± 1.09	0.47	0.018
	E	3.07 ± 0.77	3.02 ± 0.89	−0.05	0.78
Proportional Coverage Time (%)	A	18.04 ± 3.47	18.63 ± 4.65	0.14	0.66
	B	21.11 ± 3.92	20.50 ± 4.08	−0.15	0.50
	C	22.70 ± 10.47	18.74 ± 8.51	−0.42	0.10
	D	22.93 ± 9.92	28.02 ± 9.88	0.51	0.44
	E	15.23 ± 3.42	14.11 ± 4.14	−0.29	0.34

*p-values are determined by linear mixed-effects (for mean duration and frequency of occurrence) and Dirichlet (for proportional coverage time) regression models with Wald z-tests*.

## Discussion

The current study systematically examined the dynamics of brain electric microstates characterizing the resting-state high-density electroencephalograms of people suffering from Insomnia Disorder and matched healthy controls. Using a data-driven approach, we identified 5 representative microstate classes, similar to those found in previous studies. Between-group comparison showed specifically that the mean duration of class C microstates in ID is shortened, and moreover indicated that class D microstates occur more frequently in ID.

Previous investigations on resting-state EEG microstates predominantly fixed the number of microstate classes at 4 without verifying it with objective model selection criteria [reviewed in (10, 13)]. We applied the Krzanowski–Lai criterion to a hierarchical clustering procedure to determine the optimal number of microstate classes, an approach taken by some HD-EEG microstate studies (30, 37). A recent study with a large sample size used a much more complicated criterion to determine the number of clusters and found a total number of 7 microstate classes at the group level (14), among which the 5 classes with the greatest amounts of global explained variance resemble the 5 microstate classes we identified. This would suggest that the 5 topographies (labeled as microstate classes A, B, C, D, and E) indeed occurred in a sufficient proportion of the participants, whereas additional microstate clusters might be too idiosyncratic to be reliably detected with our smaller sample size. Due to the competitive fitting procedure in microstate analysis, adding or removing a microstate class may substantially affect the resulting dynamic features. Comparing results between studies that use different model orders might thus not be tenable. To address this concern, we performed additional sensitivity analysis adopting the conventional 4-class model (Supplementary Figure 1). It could be shown that different methodologies did not substantially affect the observed group differences of medium effect sizes (Supplementary Table 2). This allows us to interpret the functional significance of the current findings within the context of previous studies.

EEG microstates have been widely regarded as the “atoms” of conscious mentation (10, 13, 17). Since we did not collect data on subjective mental content, we could only speculate the functional relevance of our results by comparing with previous studies on subjective mental content in ID assessed with the ARSQ (5) and on the associations between microstate properties and ARSQ scores (19). The study by Pipinis et al. found a robust negative correlation across people between the proportional coverage time of class C microstates and somatic awareness, whereas a similar but slightly weaker negative correlation with somatic awareness was also reported for the mean duration of class C microstates (19). Further stepwise regression showed that a combination of the proportional coverage time of class C microstates and the frequency of occurrence of class D microstates optimally explained inter-individual variation in somatic awareness (19). Thus, among the dimensions of spontaneous mental content differing between people suffering from ID and people without sleep complaints (5), the current findings regarding class C and D microstates may be particularly relevant to elevated somatic awareness in ID.

Moreover, the salience network, commonly associated with microstate class C, has been implicated in a wide range of interoceptive and emotional experiences as well as in salience filtering, autonomic processing, and executive control (38–40). The fronto-parietal network, commonly associated with microstate class D, comprises systems typically involved in orienting and/or stimulus-driven shifts of attention (41, 42). It is therefore reasonable that the coordination between these networks implements key mechanisms whereby sensory stimuli arising from the body are gated into awareness. In sum, these converging lines of evidence suggest that the abnormal microstate dynamic patterns we find in people with ID could possibly underpin the heightened level of somatic awareness, which may in turn underlie their heightened somatization complaints (43–45).

Our findings appear consistent with a growing number of resting-state fMRI studies showing aberrations involving the salience and attention networks in ID (46–50). We note that resting-state fMRI and EEG microstates provide complementary insights into brain network functioning: Resting-state fMRI usually studies the correlation strength within or between networks, while EEG microstates give information about their temporal activity patterns. Caveats in light of recent research, however, need to be mentioned in regard to interpreting the neural substrates of the current results. First, there is still an ongoing debate on whether EEG microstates represent time periods during which the associated networks are activated or inhibited (13, 14, 16). Second, the above discussion has followed the majority of previous works on EEG microstates by interpreting the functional roles of the microstate classes with reference to their intra-individual BOLD correlates in distinct brain networks reported in a simultaneous EEG-fMRI study (15). On the other hand, because intra-individual and inter-individual variations in microstate properties can be driven by different mental processes (18), other neural sources might be responsible for the observed differences (14, 16).

Although TANOVA suggested significant between-group topographical differences for microstate class A, the spatial correlations between the average topographies of the two groups were high for all microstate classes (Pearson *r* = 0.996, 0.998, 0.997, 0.998, and 0.997 for microstate classes A, B, C, D, and E, respectively). To further explore the topographical differences for microstate class A, electrode-wise *t*-tests were carried out. Results indicated that group differences were mainly circumscribed to the left lateral parietal region, where the normalized absolute voltage was lower in ID. This finding might reflect subtly but systematically different network activity between the groups contributed by regional sources within a largely intact distributed network.

The stringent selection criteria the current study employed ensured that anxiety and depression symptom severity for all participants was below clinical thresholds, although it could still be observed that people with ID tended to report higher levels of anxiety and depression than CTRL. Of note, among the instruments we used to assess anxiety and depression, the BDI-IA includes items on sleep and fatigue which overlap with ID symptomology. The group differences are in line with previous studies showing that people with ID and no depression or anxiety disorders are likely to report mild levels of depression and anxiety (51, 52), and in line with recently found strong genetic correlations of insomnia with both anxiety and depression (53, 54). Curiously few studies have investigated EEG microstate alterations in depression and anxiety disorders. One study found an increased mean duration and more proportional coverage time of class A microstates as well as reduced frequency of occurrence of class C microstates in patients with panic disorder (55). These effects differ from the ones we here report for ID. An early study focused on EEG microstates in depression (56). The methodology of this study deviates substantially from contemporary microstate analysis, making the results difficult to be compared with. More research is needed in order to better disentangle how microstate properties are related to depression, anxiety, and insomnia.

EEG microstate analysis may serve as a valuable paradigm for future investigations on nocturnal mentation in ID. Previous studies have shown that insomnia severity is associated with the frequency of thought-like nocturnal mentation (57) which might be experienced as wakefulness (58). It is still unclear, however, whether (and how) distinct nocturnal thought content contributes differentially to the experience of insomnia (59, 60). While self-report provides a more direct assessment of mental content, it bears challenging methodological limitations for nocturnal mentation. Real-time reporting would interfere with the very process of sleep initiation or maintenance under study, while responses collected after a night of sleep are prone to forgetting or recall bias. In comparison, using EEG to assess the neural correlates of mentation is less disruptive and not hampered by these limitations. It awaits future investigations to validate the value of EEG microstate features and other possible neural correlates of momentary mental content in evaluating the content of nocturnal mentation, and in bridging the gaps in current understanding of ID.

## Conclusions

The current study assessed, for the first time, resting-state EEG microstate dynamics in people with Insomnia Disorder as compared to matched healthy controls. It is found that ID is especially associated with a shorter mean duration of class C microstates and more frequent occurrence of class D microstates. These microstate alterations may underlie heightened somatic awareness in ID. Properties of EEG microstates are promising objective markers of mental content and could facilitate future investigations on nocturnal mentation or the subjective experience during the transition between wake and sleep or other conditions where self-report of mental content is not possible or desirable. Addressing somatic awareness could benefit psychotherapeutic treatment of insomnia, and the development of effective strategies to do so could profit from assessment of EEG microstate properties as possible biomarkers of somatic awareness.

## Author contributions

JR, MC, and BtL collected the data. YW, MC, and BtL prepared and organized data in digital formats. YW and MC performed the analysis. EV supervised the project. YW wrote the manuscript. All authors participated in the revision of the manuscript.

### Conflict of interest statement

The authors declare that the research was conducted in the absence of any commercial or financial relationships that could be construed as a potential conflict of interest.

## Footnotes

^1^In particular, the proportional coverage time correlates mostly negatively between microstate classes, while the mean duration, as well as frequency of occurrence, correlates positively between microstate classes.

^2^We here report global explained variance calculated according to the formula given in Murray et al. (31). Studies that explicitly mentioned to have used the same formula reported a similar amount of global variance explained by the microstates (11, 30). However, when we examined the source code of a few open source software packages for microstate analysis, we found that the implementation therein deviates from this definition. The difference in definition may be one of the reasons accounting for the discrepancy between studies regarding the global variance explained by 4 microstate classes which has been recently noted (13).

## References

[1] Morin CM Drake CL Harvey AG Krystal AD Manber R Riemann D. Insomnia disorder. Nat Rev Dis Prim. (2015) 1:15026. 10.1038/nrdp.2015.26 27189779

[2] Harvey AG. A cognitive model of insomnia. Behav Res Ther. (2002) 40:869–93. 10.1016/S0005-7967(01)00061-4 12186352

[3] Espie CA Broomfield NM MacMahon KMA Macphee LM Taylor LM. The attention-intention-effort pathway in the development of psychophysiologic insomnia: a theoretical review. Sleep Med Rev. (2006) 10:215–45. 10.1016/j.smrv.2006.03.002 16809056

[4] Hiller RM Johnston A Dohnt H Lovato N Gradisar M. Assessing cognitive processes related to insomnia: a review and measurement guide for Harvey's cognitive model for the maintenance of insomnia. Sleep Med Rev. (2015) 23:46–53. 10.1016/j.smrv.2014.11.006 25645129

[5] Palagini L Cellini N Mauri M Mazzei I Simpraga S Dell'Osso L. Multiple phenotypes of resting-state cognition are altered in insomnia disorder. Sleep Health (2016) 2:239–45. 10.1016/j.sleh.2016.05.003 29073428

[6] Diaz BA Van Der Sluis S Benjamins JS Stoffers D Hardstone R Mansvelder HD. The ARSQ 2.0 reveals age and personality effects on mind-wandering experiences. Front Psychol. (2014) 5:271. 10.3389/fpsyg.2014.00271 24772097 PMC3982068

[7] Barrett LF. The future of psychology: connecting mind to brain. Perspect Psychol Sci. (2009) 4:326–39. 10.1111/j.1745-6924.2009.01134.x 19844601 PMC2763392

[8] Christoff K Irving ZC Fox KCR Spreng RN Andrews-Hanna JR. Mind-wandering as spontaneous thought: a dynamic framework. Nat Rev Neurosci. (2016) 17:718–31. 10.1038/nrn.2016.113 27654862

[9] Kucyi A. Just a thought: how mind-wandering is represented in dynamic brain connectivity. Neuroimage (2018) 10.1016/j.neuroimage.2017.07.001. [Epub ahead of print]. 28684334

[10] Khanna A Pascual-Leone A Michel CM Farzan F. Microstates in resting-state EEG: current status and future directions. Neurosci Biobehav Rev. (2015) 49:105–13. 10.1016/j.neubiorev.2014.12.010 25526823 PMC4305485

[11] Khanna A Pascual-Leone A Farzan F. Reliability of resting-state microstate features in electroencephalography. PLoS ONE (2014) 9:e114163. 10.1371/journal.pone.0114163 25479614 PMC4257589

[12] Koenig T Prichep L Lehmann D Sosa PV Braeker E Kleinlogel H. Millisecond by millisecond, year by year: normative EEG microstates and developmental stages. Neuroimage (2002) 16:41–8. 10.1006/nimg.2002.1070 11969316

[13] Michel CM Koenig T. EEG microstates as a tool for studying the temporal dynamics of whole-brain neuronal networks: a review. Neuroimage (2018) 10.1016/j.neuroimage.2017.11.062. [Epub ahead of print]. 29196270

[14] Custo A Van Der Ville D Wells WM Tomescu MI Brunet D Michel CM. Electroencephalographic resting-state networks: source localization of microstates. Brain Connect. (2017) 7:671–82. 10.1089/brain.2016.0476 28938855 PMC5736178

[15] Britz J Van De Ville D Michel CM. BOLD correlates of EEG topography reveal rapid resting-state network dynamics. Neuroimage (2010) 52:1162–70. 10.1016/j.neuroimage.2010.02.052 20188188

[16] Milz P Pascual-Marqui RD Achermann P Kochi K Faber PL. The EEG microstate topography is predominantly determined by intracortical sources in the alpha band. Neuroimage (2017) 162:353–61. 10.1016/j.neuroimage.2017.08.058 28847493

[17] Lehmann D Consciousness: microstates of the brain's electric field as atoms of thought and emotion. In: Pereira A Jr Lehmann D, editors. The Unity of Mind, Brain and World: Current Perspectives on a Science of Consciousness. Cambridge: Cambridge University Press (2013). p. 191–218.

[18] Milz P Faber PL Lehmann D Koenig T Kochi K Pascual-Marqui RD. The functional significance of EEG microstates—associations with modalities of thinking. Neuroimage (2016) 125:643–56. 10.1016/j.neuroimage.2015.08.023 26285079

[19] Pipinis E Melynyte S Koenig T Jarutyte L Linkenkaer-Hansen K Ruksenas O. Association between resting-state microstates and ratings on the Amsterdam Resting-State Questionnaire. Brain Topogr. (2017) 30:245–8. 10.1007/s10548-016-0522-2 27647317

[20] American Psychiatric Association Diagnostic and Statistical Manual of Mental Disorder 5th ed. Washington, DC: American Psychiatric Publishing (2013).

[21] Wei Y Ramautar JR Colombo MA Stoffers D Gómez-Herrero G van der Meijden WP. I keep a close watch on this heart of mine: increased interoception in insomnia. Sleep (2016) 39:2113–24. 10.5665/sleep.6308 27634787 PMC5103799

[22] Zigmond AS Snaith RP. The hospital anxiety and depression scale. Acta Psychiatr Scand. (1983) 67:361–70. 10.1111/j.1600-0447.1983.tb09716.x 6880820

[23] Beck AT Epstein N Brown G Steer RA. An inventory for measuring clinical anxiety: psychometric properties. J Consult Clin Psychol. (1988) 56:893–7. 10.1037/0022-006X.56.6.893 3204199

[24] Beck AT Steer RA Manual for the Beck Depression Inventory. San Antonio, TX: Psychological Corporation (1993).

[25] Bastien CH Vallières A Morin CM. Validation of the Insomnia Severity Index as an outcome measure for insomnia research. Sleep Med. (2001) 2:297–307. 10.1016/S1389-9457(00)00065-4 11438246

[26] Katkovnik V Egiazarian K Astola J Local Approximation Techniques in Signal and Image Processing. Bellingham, WA: SPIE Press (2006). p. 553.

[27] Iglewicz B Hoaglin DC How to Detect and Handle Outliers. Milwaukee, WI: ASQC Quality Press (1993). (The ASQC Basic References in Quality Control: Statistical Techniques Vol. 16).

[28] Jung T-P Makeig S Humphries C Lee T-W McKeown MJ Iragui V. Removing electroencephalographic artifacts by blind source separation. Psychophysiology (2000) 37:163–78. 10.1111/1469-8986.3720163 10731767

[29] Tomescu MI Rihs TA Becker R Britz J Custo A Grouiller F. Deviant dynamics of EEG resting state pattern in 22q11.2 deletion syndrome adolescents: a vulnerability marker of schizophrenia? Schizophr Res. (2014) 157:175–81. 10.1016/j.schres.2014.05.036 24962438

[30] Gschwind M Hardmeier M Van De Ville D Tomescu MI Penner I-K Naegelin Y. Fluctuations of spontaneous EEG topographies predict disease state in relapsing-remitting multiple sclerosis. NeuroImage Clin. (2016) 12:466–77. 10.1016/j.nicl.2016.08.008 27625987 PMC5011177

[31] Murray MM Brunet D Michel CM. Topographic ERP analyses: a step-by-step tutorial review. Brain Topogr. (2008) 20:249–64. 10.1007/s10548-008-0054-5 18347966

[32] R Core Team R: A Language and Environment for Statistical Computing. Vienna: R Foundation for Statistical Computing (2015).

[33] Bates D Mächler M Bolker B Walker S Fitting linear mixed-effects models using lme4. J Stat Softw. (2015) 67:1 10.18637/jss.v067.i01

[34] Gueorguieva R Rosenheck R Zelterman D. Dirichlet component regression and its applications to psychiatric data. Comput Stat Data Anal. (2008) 52:5344–55. 10.1016/j.csda.2008.05.030 22058582 PMC3207324

[35] Maier MJ DirichletReg: Dirichlet Regression for Compositional Data in R. Vol. 125, Research Report Series/Department of Statistics and Mathematics. WU Vienna University of Economics and Business (2014).

[36] Rieger K Diaz Hernandez L Baenninger A Koenig T. 15 years of microstate research in schizophrenia—where are we? A meta-analysis. Front Psychiatry (2016) 7:22. 10.3389/fpsyt.2016.00022 26955358 PMC4767900

[37] Hatz F Hardmeier M Bousleiman H Rüegg S Schindler C Fuhr P. Reliability of functional connectivity of electroencephalography applying microstate-segmented versus classical calculation of phase lag index. Brain Connect. (2016) 6:461–9. 10.1089/brain.2015.0368 27220459

[38] Medford N Critchley HD. Conjoint activity of anterior insular and anterior cingulate cortex: awareness and response. Brain Struct Funct. (2010) 214:535–49. 10.1007/s00429-010-0265-x 20512367 PMC2886906

[39] Menon V. Large-scale brain networks and psychopathology: a unifying triple network model. Trends Cogn Sci. (2011) 15:483–506. 10.1016/j.tics.2011.08.003 21908230

[40] Uddin LQ. Salience processing and insular cortical function and dysfunction. Nat Rev Neurosci. (2015) 16:55–61. 10.1038/nrn3857 25406711

[41] Vossel S Geng JJ Fink GR. Dorsal and ventral attention systems: distinct neural circuits but collaborative roles. Neuroscience (2014) 20:150–9. 10.1177/1073858413494269 23835449 PMC4107817

[42] Petersen SE Posner MI. The attention system of the human brain: 20 years after. Annu Rev Neurosci. (2012) 35:73–89. 10.1146/annurev-neuro-062111-150525 22524787 PMC3413263

[43] Hammad MA Barsky AJ Regestein QR. Correlation between somatic sensation inventory scores and hyperarousal scale scores. Psychosomatics (2001) 42:29–34. 10.1176/appi.psy.42.1.29 11161118

[44] Zhang J Lam S-P Li SX Tang NL Yu MWM Li AM. Insomnia, sleep quality, pain, and somatic symptoms: sex differences and shared genetic components. Pain (2012) 153:666–73. 10.1016/j.pain.2011.12.003 22277557

[45] Wei Y Blanken TF Van Someren EJW Insomnia really hurts: effect of a bad night's sleep on pain increases with insomnia severity. Front Psychiatry (2018) 9:377 10.3389/fpsyt.2018.00377 PMC6121188 30210367

[46] Tagliazucchi E Van Someren EJW. The large-scale functional connectivity correlates of consciousness and arousal during the healthy and pathological human sleep cycle. Neuroimage (2017) 160:55–72. 10.1016/j.neuroimage.2017.06.026 28619656

[47] Li S Tian J Li M Wang T Lin C Yin Y. Altered resting state connectivity in right side frontoparietal network in primary insomnia patients. Eur Radiol. (2018) 28:664–72. 10.1007/s00330-017-5012-8 28828546

[48] Dong X Qin H Wu T Hu H Liao K Cheng F. Rest but busy: aberrant resting-state functional connectivity of triple network model in insomnia. Brain Behav. (2018) 8:e00876. 10.1002/brb3.876 29484254 PMC5822570

[49] Li Z Chen R Guan M Wang E Qian T Zhao C. Disrupted brain network topology in chronic insomnia disorder: a resting-state fMRI study. NeuroImage Clin. (2018) 18:178–85. 10.1016/j.nicl.2018.01.012 29387533 PMC5789127

[50] Liu X Zheng J Liu B-X Dai X-J. Altered connection properties of important network hubs may be neural risk factors for individuals with primary insomnia. Sci Rep. (2018) 8:5891. 10.1038/s41598-018-23699-3 29651014 PMC5897381

[51] Carney CE Ulmer C Edinger JD Krystal AD Knauss F. Assessing depression symptoms in those with insomnia: an examination of the Beck Depression Inventory second edition (BDI-II). J Psychiatr Res. (2009) 43:576–82. 10.1016/j.jpsychires.2008.09.002 18954876 PMC2677199

[52] Carney CE Moss TG Harris AL Edinger JD Krystal AD. Should we be anxious when assessing anxiety using the Beck Anxiety Inventory in clinical insomnia patients? J Psychiatr Res. (2011) 45:1243–9. 10.1016/j.jpsychires.2011.03.011 21482427 PMC3157494

[53] Hammerschlag AR Stringer S de Leeuw CA Sniekers S Taskesen E Watanabe K. Genome-wide association analysis of insomnia complaints identifies risk genes and genetic overlap with psychiatric and metabolic traits. Nat Genet. (2017) 49:1584–92. 10.1038/ng.3888 28604731 PMC5600256

[54] Jansen PR Watanabe K Stringer S Skene N Bryois J Hammerschlag AR Genome-wide analysis of insomnia (N = 1,331,010) identifies novel loci and functional pathways. bioRxiv 214973 (2018). 10.1101/214973 30804565

[55] Kikuchi M Koenig T Munesue T Hanaoka A Strik W Dierks T. EEG microstate analysis in drug-naive patients with panic disorder. PLoS ONE (2011) 6:e22912. 10.1371/journal.pone.0022912 21829554 PMC3146502

[56] Strik WK Dierks T Becker T Lehmann D. Larger topographical variance and decreased duration of brain electric microstates in depression. J Neural Transm. (1995) 99:213–22. 10.1007/BF01271480 8579806

[57] Wassing R Benjamins JS Dekker K Moens S Spiegelhalder K Feige B. Slow dissolving of emotional distress contributes to hyperarousal. Proc Natl Acad Sci USA. (2016) 113:2538–43. 10.1073/pnas.1522520113 26858434 PMC4780629

[58] Feige B Nanovska S Baglioni C Bier B Cabrera L Diemers S. Insomnia—perchance a dream? Results from a NREM/REM sleep awakening study in good sleepers and patients with insomnia. Sleep (2018) 41:zsy032. 10.1093/sleep/zsy032 29432570

[59] Fichten CS Libman E Creti L Amsel R Sabourin S Brender W Role of thoughts during nocturnal awake times in the insomnia experience of older adults. Cognit Ther Res. (2001) 25:665–92. 10.1023/A:1012963121729

[60] Alapin I Libman E Bailes S Fichten CS. Role of nocturnal cognitive arousal in the complaint of insomnia among older adults. Behav Sleep Med. (2003) 1:155–70. 10.1207/S15402010BSM0103_3 15600219

