# Supplementary material for: EEG Microstates Indicate Heightened Somatic Awareness in Insomnia: Toward Objective Assessment of Subjective Mental Content
Source: Front Psychiatry. 2018 Sep 6;9:395. doi: 10.3389/fpsyt.2018.00395 (PMC6135918; doi:10.3389/fpsyt.2018.00395)
Supplement: Supplementary file 1 [file Data_Sheet_1.doc]

Supplementary Material

# EEG Microstates Indicate Heightened Somatic Awareness in Insomnia: Towards Objective Assessment of Subjective Mental Content

Yishul Wei, Jennifer R. Ramautar, Michele A. Colombo, Bart H.W. te Lindert, Eus J.W. Van Someren*

*** Correspondence:** Eus J.W. Van Someren: e.j.w.someren@vu.nl

**Fine-Grained Microstate Dynamics**

Because EEG scalp topography remains stable and has the highest signal-to-noise ratio at the local maxima of the global field power (GFP) (1), it is a common practice to fit microstate classes only at the GFP local maxima and subsequently interpolate class assignment to derive the dynamic features (2). Fine-grained microstate dynamics occurring between GFP local maxima are thus masked. Currently, no empirical study has investigated the practical effect of this procedure.

In parallel with our main analysis, we fitted microstate topographies to continuous EEG recordings, assigning each timeframe to a microstate class, and examined dynamic features deriving from the resulting fine-grained microstate sequences. Not surprisingly, many more apparent microstate transitions were observed, and the apparent duration of microstates calculated from the fine-grained microstate sequences was much shorter (~10–15 ms) as compared to the duration calculated from the microstate sequences with labels interpolated between GFP local maxima. Notwithstanding this limitation, the correlations between microstate dynamic features derived from the fine-grained sequences and the interpolated sequences were striking: Across participants, Spearman correlation coefficients between 0.84–0.99 were attained for all features (Supplementary Table 1).

This suggests that, at least as far as the dynamic features we studied here are concerned, the fine-grained and interpolated microstate sequences contain essentially equivalent information. This result is consistent with previous studies on the scale-free dynamics of EEG microstates (3,4). Indeed, Van De Ville et al. showed that temporal smoothing with timescales up to several seconds does not destroy information in the microstate time series (3). The finding indicates that fitting microstate topographies at the GFP local maxima is sufficient for the study of microstate dynamic features. As compared to arbitrary time samples, the GFP local maxima might have more advantages for topography fitting given their more favorable signal-to-noise ratio, such as high test-retest reliability (5).

**Conventional 4-Class Model**

We performed conventional microstate analysis with the number of clusters fixed at 4 for both individual-level and group-level k-means clustering and compared the results with those derived from the optimal 5-class model. Supplementary Figure 1 and Supplementary Table 2 present the microstate topographies and dynamic features, respectively, resulting from the conventional 4-class model. The topographies labeled as microstate classes A, B, C, and D are highly consistent with those of the corresponding classes from the optimal 5-class model. Furthermore, by treating microstate classes A, B, C, and D from the 4- and 5-class models as equivalent, one observes that group differences with medium effect sizes were preserved across the 4-class and 5-class solutions, whereas those with small effect sizes were more sensitive to model specification. This supports the comparability of our main findings with those from previous studies.

1. Pascual-Marqui RD, Michel CM, Lehmann D. Segmentation of brain electrical activity into microstates: Model estimation and validation. IEEE Trans Biomed Eng. 1995;42(7):658–65.

2. Michel CM, Koenig T. EEG microstates as a tool for studying the temporal dynamics of whole-brain neuronal networks: A review. Neuroimage. 2018; in press.

3. Van De Ville D, Britz J, Michel CM. EEG microstate sequences in healthy humans at rest reveal scale-free dynamics. Proc Natl Acad Sci U S A. 2010;107(42):18179–84.

4. Gschwind M, Michel CM, Van De Ville D. Long-range dependencies make the difference—Comment on “A stochastic model for EEG microstate sequence analysis.” Neuroimage. 2015;117:449–55.

5. Khanna A, Pascual-Leone A, Farzan F. Reliability of resting-state microstate features in electroencephalography. PLoS One. 2014;9(12):e114163.

**Supplementary Table 1.** Spearman correlation coefficients between microstate features derived from fine-grained microstate sequences and those derived from interpolated microstate sequences.

|  | Microstate class | Correlation |
| --- | --- | --- |
| Mean Duration (ms) | A | 0.887 |
|  | B | 0.909 |
|  | C | 0.918 |
|  | D | 0.913 |
|  | E | 0.835 |
| Frequency of Occurrence (1/s) | A | 0.901 |
|  | B | 0.887 |
|  | C | 0.938 |
|  | D | 0.956 |
|  | E | 0.886 |
| Proportional Coverage Time (%) | A | 0.949 |
|  | B | 0.936 |
|  | C | 0.984 |
|  | D | 0.986 |
|  | E | 0.950 |

**Supplementary Table 2.** Microstate dynamic features (mean ± standard deviation) derived from the conventional 4-class model.

|  | Microstate class | Control  (*n* = 32) | Insomnia disorder  (*n* = 32) | Cohen’s *d* | *p* |
| --- | --- | --- | --- | --- | --- |
| Mean Duration (ms) | A | 55.85 ± 8.24 | 52.45 ± 8.62 | –0.40 | 0.35 |
|  | B | 56.69 ± 10.06 | 52.67 ± 7.73 | –0.45 | 0.26 |
|  | C | 67.41 ± 19.28 | 58.77 ± 14.12 | –0.51 | 0.0092 |
|  | D | 64.19 ± 11.77 | 66.36 ± 16.55 | 0.15 | 0.40 |
| Frequency of Occurrence (1/s) | A | 3.85 ± 0.97 | 4.00 ± 0.88 | 0.16 | 0.66 |
|  | B | 3.93 ± 0.75 | 3.99 ± 0.98 | 0.06 | 0.94 |
|  | C | 4.35 ± 1.05 | 4.39 ± 1.18 | 0.04 | 0.99 |
|  | D | 4.37 ± 1.41 | 4.95 ± 1.05 | 0.46 | 0.038 |
| Proportional Coverage Time (%) | A | 21.01 ± 3.88 | 20.84 ± 5.21 | –0.04 | 0.24 |
|  | B | 21.92 ± 4.28 | 20.67 ± 4.79 | –0.28 | 0.18 |
|  | C | 29.18 ± 10.40 | 25.79 ± 8.68 | –0.35 | 0.090 |
|  | D | 27.89 ± 9.42 | 32.70 ± 9.99 | 0.50 | 0.82 |

*p*-values are determined by linear mixed-effects (for mean duration and frequency of occurrence) and Dirichlet (for proportional coverage time) regression models with Wald *z*-tests.


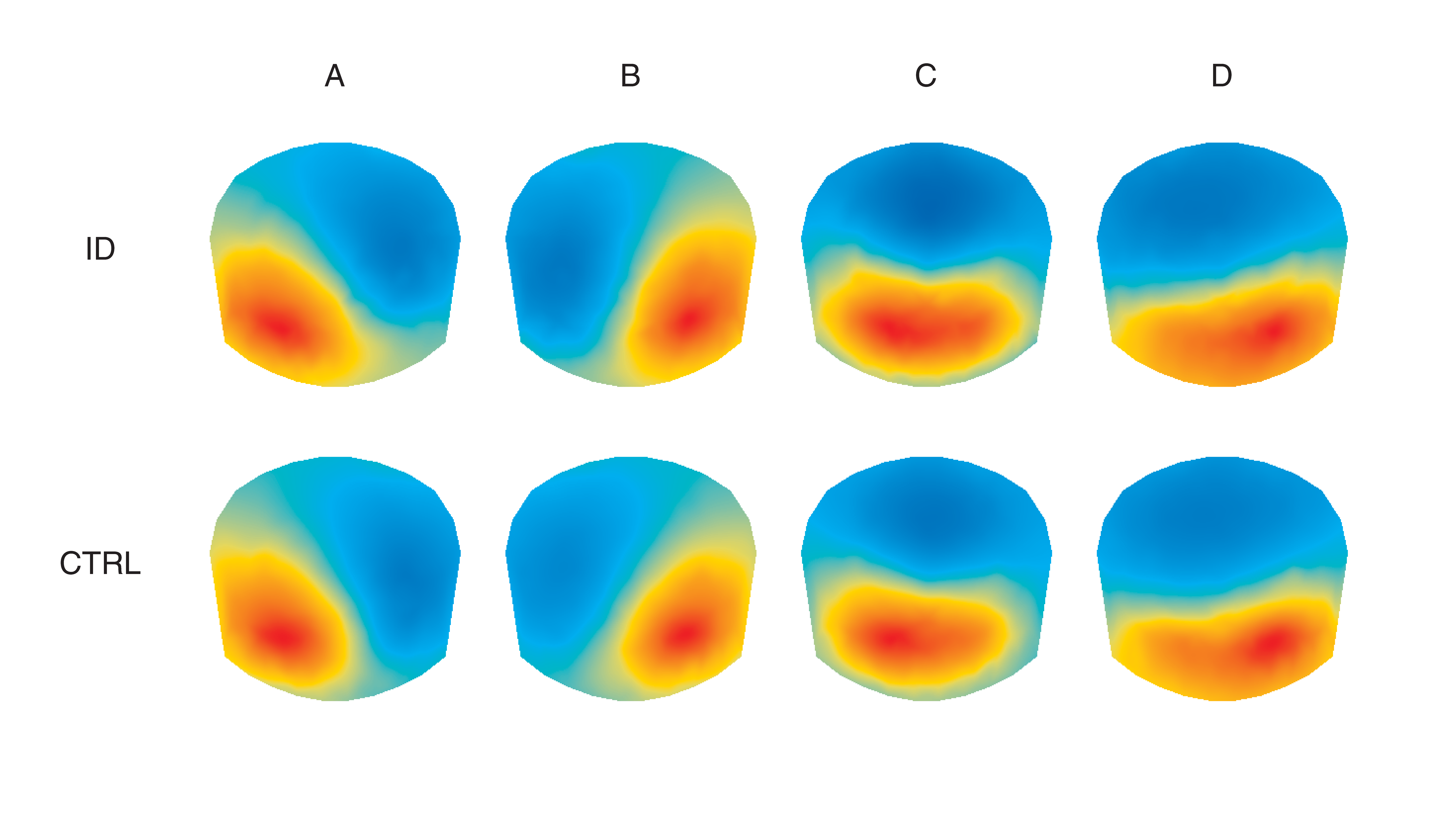


**Supplementary Figure 1.** Average topographic maps for 4 microstate classes (A, B, C, and D) in people with Insomnia Disorder (ID) and healthy controls (CTRL) resulting from the conventional 4-class model. Note that by convention microstate labeling only depends on the spatial configuration while the absolute voltage and polarity are ignored.
